# Correction: Li et al. *S100A4* Promotes BCG-Induced Pyroptosis of Macrophages by Activating the NF-κB/NLRP3 Inflammasome Signaling Pathway. *Int. J. Mol. Sci.* 2023, *24*, 12709

**DOI:** 10.3390/ijms26199406

**Published:** 2025-09-26

**Authors:** Mengyuan Li, Yueyang Liu, Xueyi Nie, Boli Ma, Yabo Ma, Yuxin Hou, Yi Yang, Jinrui Xu, Yujiong Wang

**Affiliations:** 1 School of Life Sciences, Ningxia University, Yinchuan 750021, China; 20180044@nxmu.edu.cn (M.L.); 18395273708@163.com (Y.L.); 18794898774@163.com (X.N.); 15695013087@163.com (B.M.); myb512816@163.com (Y.M.); 18209689779@163.com (Y.H.); yangyi@nxu.edu.cn (Y.Y.); 2 Key Laboratory of Ningxia Minority Medicine Modernization, Ministry of Education, Ningxia Medical University, Yinchuan 750004, China

In the original publication [1], there was a mistake in Figure 8F as published. The Western blotting bands for ASC and Caspase-1 exhibit unintended duplication. The corrected Figure 8 appears below. The authors state that the scientific conclusions are unaffected. This correction was approved by the Academic Editor. The original publication has also been updated.

## Figures and Tables

**Figure 8:**
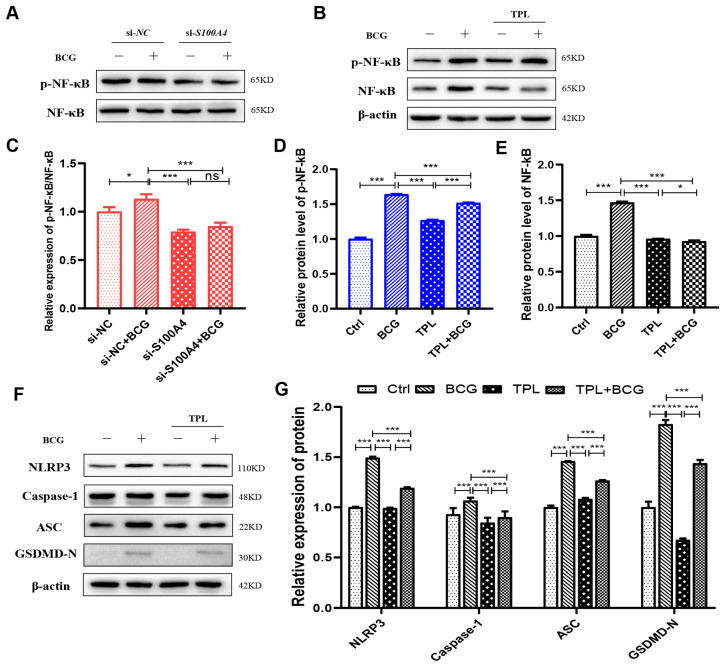
Knockdown of *S100A4* inhibited the NF-κB signaling pathway and reduced the expression of NLRP3 inflammasome and pyroptosis-related proteins. (**A**,**B**) si-*S100A4* downregulated the expression of p-NF-κB. The protein expression levels of p-NF-κB and NF-κB were analyzed using Western blot in the THP-1 macrophages pre-treated with si-*S100A4* for 24 h and infected with BCG for 24 h subsequently. (**C**–**E**) The protein expression levels of p-NF-κB and NF-κB in the THP-1 macrophages, which were pre-treated with Triptolide (TPL) 5 nM for 2 h and infected with BCG for 24 h, were quantitatively analyzed using Western blot. (**F**,**G**) Inhibition of NF-κB decreased the expression of inflammatory-related proteins (NLRP3, Caspase-1 p48, ASC) and a pyroptosis-related protein (GSDMD-N). Data are from mean ± SD of three independent experiments, ^ns^ *p* > 0.05, * *p* < 0.05, *** *p* < 0.001.
